# Harnessing plant microbiomes to enhance crop resilience and restore war-affected soils in Ukraine

**DOI:** 10.3389/fpls.2026.1868751

**Published:** 2026-06-30

**Authors:** Sergiy Shulga, Olena Tigunova, Hanna Andriiash, Alla Yemets, Yaroslav Blume

**Affiliations:** 1 Laboratory of Industrial and Food Biotechnology, Institute of Food Biotechnology and Genomics, National Academy of Sciences of Ukraine, Kyiv, Ukraine; 2 Department of Cell Biology and Biotechnology, Institute of Food Biotechnology and Genomics, National Academy of Sciences of Ukraine, Kyiv, Ukraine; 3 Department of Genomics and Molecular Biotechnology, Institute of Food Biotechnology and Genomics, National Academy of Sciences of Ukraine, Kyiv, Ukraine

**Keywords:** bioremediation, metagenome, microbial engineering, plant stress, rhizosphere microbiome, soil fertility, war-related pollution

## Abstract

This review presents the current understanding of the rhizosphere microbiome and its potential application for the regeneration of damaged soils. The aim was to examine the issues of soil degradation associated with military actions and the latest developments in microbiome engineering for their application in the bioremediation of damaged lands. The review analyses recent developments and achievements in the study of the microbiome, its role in soil fertility, and plant protection against stress. Various directions and approaches to microbial profiling and addressing relevant pollution issues using developed bioengineered models and constructs have been examined. It has been shown that the most common explosive organic compounds — TNT, hexogen, and octahydro-1,3,5,7-tetranitro-1,3,5,7-tetrazocine — and heavy metals — lead, cadmium, zinc, and antimony — account for the greatest soil contamination. The restoration of soils damaged as a result of military actions is feasible through the engineering of a specific soil microbiome (including genera *Bacillus*, *Pseudomonas*, and *Arthrobackter*, as well as arbuscular mycorrhiza). Military-related stress on soil is exerted by a mixture of organic pollutants and heavy metals, and the use of microbial consortia is a promising approach for mitigating their impact. The main economic advantage of such associations is that a consortium not only degrades toxic contaminants but also contains strains capable of nitrogen fixation and phosphorus mobilisation. The economic feasibility of applying synthetic microbial consortia and microbial engineering in war-affected regions is based on balancing the initial costs of research and development against substantial savings in capital investments compared with conventional land remediation methods.

## 1 Introduction

Military conflicts cause long-term destructive effects on the environment, including soil degradation, toxic contamination, habitat destruction, and significant carbon emissions. Modern warfare, such as the conflict in Ukraine, devastates ecosystems through the use of heavy machinery, chemical leakages, and unexploded ordnance, leading to mass contamination (Levy, 2025). Military conflicts bring about a multifaceted negative impact on landscapes, resulting in deforestation, the contamination of water resources and soils, the loss of biodiversity, and the degradation of air quality caused by explosions, fires, and the use of toxic substances. Military operations devastate protected areas and forest tracts, while also leading to the pollution of agricultural lands and the generation of significant amounts of hazardous waste (Wirtu and Abdela, 2025). Bombardments and artillery strikes leave behind contamination in the form of heavy metals and other toxic substances. These substances penetrate soil and water, threatening not only flora and fauna but also human health.

Given the identified environmental threats, there is an urgent need to develop and implement comprehensive strategies for monitoring, assessing, and restoring natural landscapes in the post-war period. These strategies must envisage modern scientific approaches, innovative technologies, and international experience in environmental remediation (Drebot and Korol, 2025). Following a shell explosion, heavy metals enter the soil, posing a moderate level of environmental risk, altering the physical and chemical properties of the soil, reducing bacterial diversity, and shifting the taxonomic structure of the mocrobiome (Maslovska et al., 2025). Nature can recover on its own, yet it is difficult to predict how long this process might take. The primary environmental consequences of the war for Ukraine include desertification and the challenge of accessing water resources; the contamination of water bodies, rendering them unfit for use; shifts in flora and fauna, the loss of forest cover, declining biodiversity, and the survival of only the most resilient species; the loss of soil fertility, along with the contamination and burning of the fertile topsoil as a result of military operations; and the emergence of hazardous areas with lands contaminated by explosives, biologically pathogenic agents, and chemically toxic substances (Margitay et al., 2025). This review aimed to investigate modern methods and developments in microbiome engineering to address the outlined soil-related challenges. The research methodology consisted of analysing and summarising information from scientific literature sources such as international databases, including Scopus and Web of Science, as well as major biomedical and life science databases such as PubMed and the full-text journal platform ScienceDirect. The literature review focused on the role of the microbiome in both healthy soils and soils contaminated due to military activities, including both the direct effects of explosions and chemical contamination with heavy metals and residues of explosive compounds. To select literature sources in international databases, the search query was formed from the terms microbiome, soil, military operations, metagenomic research, degradation, pollutants, remediation and their combinations. The criteria for selecting articles included works that investigated changes in the structure, diversity (quantitative and qualitative composition) and functions of the soil microbiome. For literature selection in international databases, search queries were constructed using the terms microbiome, soil, military activities, metagenomic analysis, degradation, pollutants, remediation, and their combinations. The inclusion criteria comprised studies investigating changes in the structure, diversity (both qualitative and quantitative composition), and functions of the soil microbiome. Particular attention was given to publications addressing the impacts of military-related factors, including shelling, explosions, military vehicles, fires, and contamination by heptyl, trinitrotoluene (TNT), hexogen (RDX), and heavy metals (Pb, Cu, Zn, and Cd). Studies evaluating bioremediation approaches and the restoration of war-affected soils using microorganisms were also included. The literature search covered all available publication years to provide historical context, with a primary focus on studies published within the last decade.

## 2 State of land contamination during military actions

**Figure 1:**
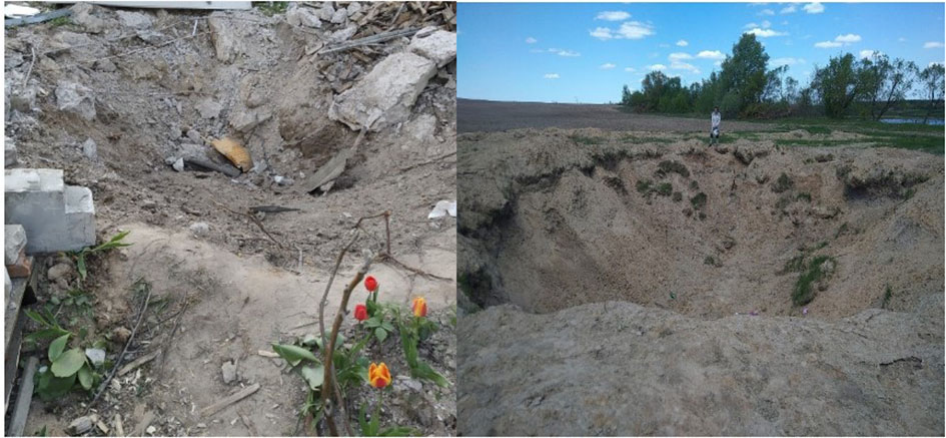
Soil damage due to bombing in the Kyiv region.

Active military operations, which took place in the course of a full-scale armed invasion of the Russian Federation into the territory of Ukraine, have led to substantial soil damage and destruction (Kucher, 2025). As of 2026, approximately one-third of Ukraine’s arable land has been degraded due to military actions (Kazak et al., 2026). The militarisation-induced impact on soils can be categorised into chemical (contamination with heavy metals and explosives), mechanical (physical destruction of soil layers and humus), and biological (degeneration or intentional contamination with microorganisms harmful to living organisms). The assessment of the damage caused to land and soils as a result of emergencies and/or armed aggression and military actions during martial law is determined in accordance with the methodology approved by Order No. 167 of the Ministry of Environmental Protection and Natural Resources of Ukraine, dated April 4, 2022. Debris of destroyed military equipment, ammunition, and fuel residues leads to multifaceted damage to the soil system, causing local and global contamination as well as the loss of soil resources (**Figure 1**).

Numerous consequences of military operations in Ukraine demand close attention. In particular, it is crucial to investigate their impact on the physical (particle-size distribution), chemical (heavy metal concentrations), and biological (soil microbiome and mesofauna) characteristics of disturbed soil systems. Soil contamination with explosives can disrupt microbial communities, leading to shifts in biodiversity. Recent studies have shown that the impact of ammunition significantly reduces the diversity and richness of soil microbes, as evidenced by declines in diversity indices such as the Shannon index (Flores et al., 2025).

The impact on the soil ecosystem caused by explosions and the pyrolysis of armoured vehicles has led to a significant increase in the abundance of micromycetes, pedotrophs, and oligotrophs, indicating contamination with various organic and inorganic toxic compounds. These stressful conditions trigger active adaptation of microorganisms and their involvement in the transformation of organic residues. Chemical and physical soil degradation at explosion sites has manifested as changes in particle size fractions within blast zones, combustion areas, and sites contaminated with heavy metals (HMs). The combustion of military equipment led to a 1.2–1.8-fold increase in the sand fraction and a 1.1–1.2-fold decrease in the clay fraction. Soil contamination with HMs significantly exceeded sanitary norms, with the highest levels of pollution detected for Pb, Zn, and Cd. In all affected areas, shifts in the microbiome structure occurred (with a 20-fold increase in the proportion of mycelial organisms), while the activity of microbiological processes decreased by 1.2 times, and microbial biomass declined by 2.1 times. It has been established that soils within military sites remain significantly contaminated with toxic compounds from ammunition and their residues for many decades, containing poisonous metals—antimony (Sb), lead (Pb), and uranium (U)—as well as chemical substances such as 2,4-dinitrotoluene, 2,4,6-trinitrotoluene (TNT), 1,3,5-trinitro-1,3,5-triazacyclohexane (RDX), among others (Solokha et al., 2024). Elevated concentrations of heavy metals in the soil not only cause direct toxic effects but also indirectly negatively impact the reproduction and bioproduction of soil microorganisms. Most of these compounds are resistant to biodegradation or conventional treatment; thus, they persist in the biosphere, serving as a continuous source of contamination and toxic impact, potentially harming human health and the environment. When assessing the impact of potentially toxic substances on the soil system resulting from military actions, the primary focus is placed on the study of bacterial communities. Major research efforts have been directed toward identifying active microorganisms to facilitate bioremediation of contaminated soils. The high toxicity of explosive compounds, combined with the stability of their chemical structures and their ability to bind with soil organic matter, hinders and significantly complicates soil restoration efforts (Robinson et al., 2024). Rewilding requires a detailed study of the impact consequences and the use of a comprehensive approach to overcome them, involving all available techniques, approaches, and methods — the first step of which is the study of the soil microbiome.

Military activities cause both direct and indirect damage to soil ecosystems. Explosions release heavy metals, such as lead, cadmium, and arsenic, as well as residues of explosive compounds, including trinitrotoluene (TNT) and hexogen (RDX). Heavy military machinery and explosive impacts disrupt soil structure, leading to erosion and land degradation (Melnychuk et al., 2026). Large areas of land are removed from economic use due to the danger of landmines. In addition, fires at fuel storage facilities and the destruction of industrial infrastructure contaminate soils with a wide range of hazardous chemicals. Information regarding the specific characteristics and extent of soil contamination resulting from military activities remains either unavailable or rather limited (Didenko, 2024). Research on the migration of modern explosive compounds and their degradation products in different soil types remains extremely limited. There is also a lack of data regarding the combined effects of multiple toxic contaminants on soil microbiomes. The exact degradation rates and persistence of certain military-related chemicals under natural environmental conditions are still poorly understood. Furthermore, comprehensive modelling of the transfer of toxic substances from soil into crops is required to assess potential risks to food safety and human health. Damage assessment in conflict-affected areas is further complicated by significant technical and security challenges. In many cases, soil sampling is impossible in mine-contaminated areas or in territories located near active frontlines. These limitations hinder environmental monitoring efforts and create substantial gaps in our understanding of the long-term ecological consequences of military activities. Satellite images capture craters and fires, and other visible signs of military activity; however, it provides no information regarding the type, concentration, and distribution of toxic substances in soils. In addition, reliable pre-war soil data are often unavailable, making accurate “before-and-after” comparisons difficult or impossible. Existing national standards and international monitoring protocols were not designed to assess the specific chemical compounds and complex contaminant mixtures generated during intensive military operations. Existing national standards and international monitoring protocols were not designed to assess the specific chemical compounds and complex contaminant mixtures generated during intensive military operations. As a result, current assessment frameworks may be insufficient for accurately characterising the environmental impacts of armed conflicts, highlighting the need for the development of specialised methodologies and monitoring approaches tailored to war-related contamination.

## 3 Study of the soil microbiome

The consortium of microorganisms in every environment is unique, but soils are considered home to all the most complex and diverse microbiomes on our planet (Pandey and Saharan, 2025). A microbiome is a community of microorganisms living in a specific environment, characterised by microbial structures and substances (Iqbal et al., 2025). The soil microbiome is a complex system composed of several functional groups (Burrill et al., 2025).There are several approaches to studying the soil microbiome, which can be broadly classified into two main groups: ecological and molecular approaches (Fujita et al., 2025). Ecological approaches, utilising advanced methods for assessing fertility, ecosystem status, and the impact of anthropogenic factors, aim to provide the most comprehensive understanding of the soil microbiome state and its interaction with the environment (Gahlot, 2026).

The ecological approach includes indirect methods for evaluating the microbial community and its functions. Examples include soil respiration, plant bioassays, and the use of chemical or physical soil properties as indicators or biological markers, such as the presence of mycorrhizal fungi (Onet et al., 2025). Microbial isolation methods, where microorganisms are cultivated outside their natural environment, are also considered ecological approaches. One such approach is the use of Biolog^®^ EcoPlates™ (Biolog Inc., USA), designed to analyse the functional diversity of bacterial communities by measuring their ability to oxidise carbon substrates. Unfortunately, Biolog^®^ EcoPlates™ analysis results are sometimes difficult to reproduce and standardise due to a lack of detailed procedures and protocols (Decena and dela Cruz, 2024). The reduction in sequencing costs and technological advancements have facilitated the widespread adoption of the molecular approach (Barbosa et al., 2025). This approach involves the use of molecular biology methods to analyse the genetic material of the soil microbiome and determine its composition (Gómez-Godínez et al., 2025). It is important to note that the molecular approach, based on DNA sequencing, provides information regarding the relative abundance of microbes or genes rather than their absolute values (Pace et al., 2026). There are also other limitations regarding the capabilities of the molecular approach (Tekgul and Adiguzel, 2025). For instance, the use of molecular methods can provide misleading information about microbial presence during DNA sequencing if relic DNA is detected in the soils. This can inflate diversity estimates for bacteria and archaea by more than 40% (Sun and Ge, 2023). Sequencing errors in PCR analysis during the characterisation of soil microbial diversity can provide incorrect (inflated) information regarding the number of species (Ece et al., 2026). Conversely, sequencing errors can also lead to underestimation if databases (or their samples) are incomplete (DeFord and Yoon, 2024). Most soil taxa (i.e., microbes grouped by similar characteristics) have not yet been described and cannot be found in reference databases (Wu et al., 2025). Other challenges involve integrating multi-omics data to identify functional traits, addressing spatio-temporal variability in microbial dynamics, deciphering the interactions between plant genotypes and microbial communities, and ensuring standardised controls, metadata, depth targets, and workflow reproducibility (Benoit et al., 2026). These challenges encompass specific limitations in sequencing technologies (standardisation and reproducibility); data integration and analysis; limited functional characterisation of microbiome members; host specificity and the dependence of complexes on environmental context; achieving stability, resilience, and predictability of engineered microbiomes; the impact of coevolution and domestication on plant-microbe interactions; the translation of research into field applications; regulatory and ethical considerations; and the economic feasibility of microbiome-based interventions (Fadiji et al., 2025). Although a wide range of microbiome characteristics remains unexplored, the microbiome plays a critical role in ensuring soil functionality within agricultural production for the well-being of plants, animals, and humans. Microbiome biodiversity is typically divided into four main groups, ranked from most common to least common: bacteria, fungi, archaea, and protists (Iqbal et al., 2025). Some classifications include viruses among microbes, while others view viruses as part of the “floating genetic material” that can be studied through metagenomic analysis (Rodriguez del Rio et al., 2025). Metagenomics also opens up the possibility of studying root microbiomes (Fonseca-Garcia et al., 2025). A pronounced compositional divergence between rhizospheric and soil microbiomes—highlighting the influence of plant species and soil types on the formation of niche-specific microbial communities and functional groups—has been identified using metagenomic sequencing and bioinformatics of root-associated microbial consortia, followed by systematic classification and cataloguing (Jalal and Alshehrei, 2026).

The role of the soil microbiome in healthy soil has been increasingly confirmed over recent decades (Neale et al., 2024). Bacteria are the most prevalent microorganisms, accounting for 70–90% of the total soil biomass. Soil, containing up to 10¹¹ microorganisms per gram, serves as a dynamic interface where abiotic and biotic components interact to shape plant productivity (Liu et al., 2025). The most prevalent classes of bacterial phyla and fungi were *Acidobacteria, Actinobacteria, Armatimonadetes, Bacteroidetes, Firmicutes, Planctomycetes, Proteobacteria, Verrucomicrobia*, and *Agaricomycetes*. Furthermore, the dominant bacterial and fungal taxa across all soil layers belonged mainly to *Acidobacteria* and *Agaricomycetes*, respectively, highlighting the importance of soil layers in shaping microbial interactions (Qiao et al., 2025). Microorganisms interact with the plant, establishing a symbiotic association in the narrow zone of soil surrounding the root—the rhizosphere. This area hosts a concentrated portion of food chains and plant disease defence systems (Ren et al., 2026). It is known (Narkhede et al., 2026) that plant-associated microorganisms can synthesise phytohormones essential for symbiotic relationships. The production of these substances is a vital property of rhizospheric, epiphytic, and symbiotic bacteria (Rios-Ruiz et al., 2025). In the root zone of non-leguminous plants, the rhizosphere is composed of microorganisms from various taxonomic groups: *Acetobacter, Agrobacterium, Alcaligenes, Azoarcus, Azomonas, Azospirillum, Azotobacter, Bacillus, Clostridium, Derxia, Herbaspirillum, Enterobacter, Erwinia, Klebsiella, and Pseudomonas* (Nosheen et al., 2021). Certain taxa, including *Actinobacteria*, *Acidobacteria*, and the fungal genus *Mortierella*, consistently act as significant predictors of ecosystem multifunctionality (Romero et al., 2025). The interaction between plants, soil, and microorganisms is a complex, continuous, and dynamic process. The main functional groups of soil microorganisms and their ecological roles are presented in **Table 1**.

**Table 1:** Main functional groups of soil microorganisms and their ecological roles.

Functional group	Key processes	Main enzymes/mechanisms	Typical genera (examples)	Ecological and practical significance
Nitrogen-fixing	Atmospheric nitrogen fixation	Nitrogenase	*Rhizobium, Bradyrhizobium, Azotobacter, Azospirillum*	Providing plants with nitrogen reducing the need for mineral fertilisers
Ammonifying	Mineralisation of organic nitrogen and NH_4_^+^	Protease, deaminase	*Bacillus, Proteus, Pseudomonas*	Maintaining nitrogen availability in the soil
Nitrifying	Oxidation of NH_4_^+^ to NO_3_^-^	Ammonia and nitrite oxidase	*Nitrosomonas, Nitrobacter*	Formation of nitrite nitrogen, impact on soil acidity
Denitrifying	Reduction of NO_3_^-^ to N_2_/N_2_O	Nitrate and nitrite reductases	*Pseudomonas, Paracoccus*	Nitrogen balance regulation, greenhouse gas emissions
Phosphate-mobilizing	Solubilization of inorganic phosphates	Organic acids, chelation	*Bacillus, Pseudomonas, Enterobacter*	Increasing the bioavailability of phosphorus
Phosphate mineralizing	Hydrolysis of organic forms of phosphorus	Phosphatases, phytases	*Bacillus, Penicillium*	Involvement of organic phosphorus in the cycle
Cellulose-destructive	Cellulose destruction	Cellulases	*Cellulomonas, Trichoderma*	Mineralisation of plant residues
Lignin-destroying	Lignin destruction	Ligninases, laccases	*Basidiomycetes, Streptomyces*	Formation of humus compounds
Sulfur-oxidizing	Oxidation of reduced sulfur compounds	Sulfide oxidases	*Thiobacillus, Beggiatoa*	Regulation of sulfur availability
Sulfate-reducing	Reduction of SO_4_^2-^ to Н_2_S	Sulfate reductases	*Desulfovibrio*	Anaerobic processes, impact on metal mobility
Iron- and manganese-transforming	Oxidation/Reduction of Fe, Mn	Redox enzymes	*Geobacter, Gallionella*	Mobility of metals and toxicants
Rhizosphere PGPR	Plant growth stimulation	Sidephores, indole-3-acetic acid, deaminase	*Pseudomonas, Bacillus, Azospirillum*	Increasing plant productivity
Symbiotic	Symbiotic plant nutrition	Mycorrhiza, nodules	*Rhizobium, Glomeromycota*	Improving the absorption of nitrogen, phosphorus, and water
Biodestructors (bioremediation)	Decomposition of xenobiotics	Oxidases, dehydrogenases	*Rhodococcus, Sphingomonas, Bacillus, Acitomycetes, Acinetobacter*	Soil pollution cleanup

Rhizospheric bacteria possessing a set of plant-beneficial properties are referred to as PGPR (Plant Growth-Promoting Rhizobacteria). They are characterised by their ability to fix molecular nitrogen, synthesise auxins, gibberellins, cytokinins, vitamins, as well as antibiotic and antifungal compounds, mobilise phosphates, and degrade harmful chemical substances (Zhang et al., 2024). The production of gibberellins is characteristic of epiphytic and rhizospheric bacteria, including *Agrobacterium*, *Azotobacter*, *Azospirillum*, *Bacillus*, *Clostridium*, *Flavobacterium*, and *Pseudomonas* (Chen et al., 2024). Microbial diversity in soil is considered important for soil quality management, as it exerts various effects that stimulate plant growth or enable biocontrol (Wilharm et al., 2026), which may be beneficial for the host plant and can alter plant physiology and nutrition.

From the perspective of specific mechanisms, bacteria and fungi promote plant colonisation through the decomposition of soil organic matter, solubilization of elements such as nitrogen, phosphorus, and potassium, symbiosis with plants, secretion of phytohormones, and by influencing soil structure, development, and plant growth (Lee et al., 2025). In practical applications, beneficial microbial communities can be used to remediate contaminated soil and water, thereby reducing environmental pollution (Halo et al., 2026). It has been shown (Rodriguez et al., 2025) that *Pseudomonadota* and *Actinomycetota* dominated the overall transcriptional profile and specific functions associated with the early stages of soil development. Plants have developed epigenetic networks that regulate beneficial plant–microbe interactions by modulating immune responses, gene regulation, and metabolite production to enhance stress tolerance and adaptation to soil conditions (Tian et al., 2026). The identification of new microbial species and the prediction of their functions remain the greatest opportunities and challenges in the modern field of microbial environmental remediation (Li et al., 2026).

**Figure 2:**
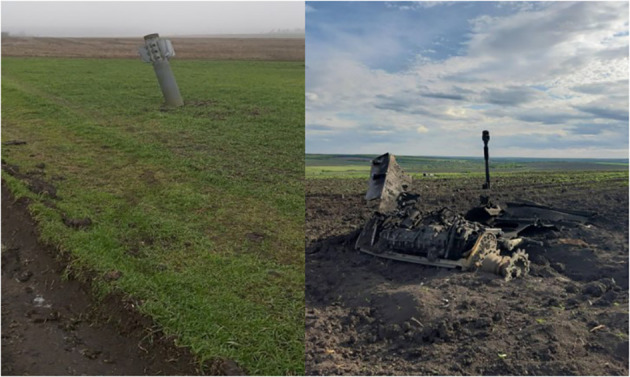
Remains of military equipment and missiles in the Kyiv region.

The study of soil microbiomes, including bacteria, archaea, fungi, and actinomycetes, in war-affected areas is critically important because these microorganisms are responsible for biogeochemical cycling, soil fertility, and the natural detoxification of contaminants. Military activities impose severe stress on soil biota, causing profound alterations in microbiome structure. A substantial decline in microbial biomass, averaging approximately twofold, as well as a reduction in the overall activity of beneficial microorganisms, has been documented. Particularly affected are nitrogen-fixing and phosphate-solubilising bacteria, along with beneficial streptomycetes (Biliavska et al., 2024). Explosions and the pyrolysis of fuels and lubricants result in an abnormal increase in the abundance of filamentous fungi, in some cases exceeding twentyfold relative to background levels. Many of these fungi are considered opportunistic phytopathogens or producers of toxic metabolites. For example, fuel leakage stimulates the proliferation of hydrocarbon-degrading bacteria but can dramatically reduce the abundance of *Azotobacter* to as little as 5% of its normal population size. The release of battery acids into soil may eliminate sensitive groups of prokaryotes. In addition, heavy military vehicles and blast waves disrupt soil structure and reduce pore space, thereby limiting oxygen availability within the soil matrix (**Figure 2**). This impairs aerobic microbial processes, particularly nitrification, while promoting denitrification and the production of greenhouse gases.

Present-day environmental microbiology lacks sufficient data to accurately predict the response and long-term dynamics of soil microbiomes following military activities. There is a significant knowledge gap regarding which indigenous bacterial strains are capable of effectively degrading the complex mixtures of contaminants generated during armed conflicts, including combinations of hexogen (RDX), pentaerythritol tetranitrate (PETN), trinitrotoluene (TNT), and white phosphorus compounds. Likewise, the behaviour of soil microbiomes under the simultaneous influence of heavy metals, explosive residues, and pH alterations remains poorly understood.

Medical waste, pharmaceutical residues left in conflict zones, and prolonged environmental stress may further promote the horizontal transfer of antibiotic resistance genes (ARGs) among soil bacteria, posing a potential threat to biosafety. In addition, the role of soil microbiomes in the mobilisation and transformation of iron and other metals released from munitions fragments has received limited attention. Nevertheless, existing evidence suggests that microbially produced organic acids can accelerate iron dissolution by up to 120-fold, converting relatively stable metal forms into more mobile and potentially toxic species (Zghurska et al., 2025). The study of soil microbiota is complicated by both the unique challenges associated with military activities and the inherent limitations of current analytical methods. Samples intended for DNA-based profiling techniques, including metagenomic analyses, require immediate preservation, typically through flash-freezing in liquid nitrogen or storage at −80°C. However, logistical constraints in active combat zones and frontline regions often make compliance with these requirements impossible, potentially compromising sample integrity and introducing bias into the results. Traditional culture-based methods, such as cultivation on nutrient media in Petri dishes, capture less than 1% of the actual diversity of soil bacterial communities. Although advanced molecular approaches, including Next-Generation Sequencing (NGS) and polymerase chain reaction (PCR)-based analyses, provide a more comprehensive assessment of microbial diversity and function, their application is often restricted by high costs and the limited availability of specialised laboratories. Furthermore, laboratory experiments can hardly replicate the extreme conditions generated by explosions, including sudden exposure to high temperatures and pressure waves capable of instantly sterilising crater soils. Soil microbiomes are also highly dynamic and respond rapidly to seasonal fluctuations, moisture availability, and temperature changes. The absence of reliable pre-war baseline data for specific agricultural fields further complicates efforts to distinguish war-induced alterations from natural temporal variation in soil microbial communities.

## 4 Functional roles of the soil microbiome

The soil microbiome plays an important role in maintaining soil quality, soil ecosystem stability, and plant productivity. PGPR, which includes bacteria, fungi, and other microorganisms, are increasingly recognised as an important factor promoting environmentally friendly agricultural practices (Ali and Moon, 2025).

### 4.1 Nutrient capture and cycling

Bioinoculants are living preparations containing effective nitrogen-fixing, phosphate-solubilising, or plant growth-promoting, environmentally friendly strains (Atkin et al., 2026). They are applied to seeds, seedlings, plant roots, or directly to the soil to enhance nutrient availability and crop productivity through bioenhancers, reduce disease incidence via antagonistic activity, and increase soil organic carbon content, thereby supporting soil quality and sustainability (Sharma et al., 2025). Pre-sowing inoculation of seeds with PGPR genera *Agrobacterium, Azospirillum, Azotobacter, Bacillus, Clostridium*, and *Flavobacterium* significantly stimulates seed germination and sprouting, as well as plant growth and yield. PGPR genera *Agrobacterium, Azospirillum, Azotobacter, Bacillus, Clostridium, Enterobacter, Flavobacterium*, and *Klebsiella* are used for the inoculation of various crops (Babar et al., 2024). However, inoculation with *B. subtilis* led to the enrichment of beneficial taxa such as *Pseudomonas, Pseudorhizobium, Pseudallescheria*, and *Chaetomium*, which can act as helper microbes supporting nutrient cycling. These results suggest that *B. subtilis* functions as a microbiome inducer, working synergistically with helper microorganisms (Kabir et al., 2026). The effects of individual PGPR strains and synthetic microbial communities (SynCom) on growth promotion were studied through phenotypic traits, differential gene expression, and key metabolic pathways. Among 59 PGPR strains representing genera such as *Bacillus, Pseudomonas, Burkholderia* sp., *Curtobacterium pusillum, Acidovorax, Sphingobium, Mitsuaria, Bacterium, Rhodanobacter, Variovorax, Ralstonia, Brevibacillus, Terrabacter, Flavobacterium, Comamonadaceae, Achromobacter, Paraburkholderia*, and *Massilia*, the most promising were *Burkholderia* sp. A2, *Pseudomonas* sp. C9, *Curtobacterium pusillum* E2, and *Bacillus velezensis* F3. This resulted in increased root length, fresh shoot biomass, dry shoot mass, number of branches, and number of root tips in the seedlings (Yu et al., 2025). Unfortunately, the current level of knowledge is still far from enabling the full realisation of their potential and the successful application of such biotechnological methods (Delgado-Baquerizo et al., 2025). These results highlight the potential of PGPR as environmentally friendly alternatives to chemical fungicides and pave the way for the development of bioinoculants (Taoussi et al., 2026). Seed biopriming with beneficial microbes is one of the cheapest and most environmentally friendly methods of enhancing seed growth, leading to rapid, uniform, and high establishment and yield. Studies have shown positive results in terms of germination rate and seedling survival (Anjali et al., 2026).

The rhizosphere and endophytic bacterial communities were investigated using 16S rRNA gene amplicon sequencing. These findings highlight the functional potential of bacteria and suggest promising candidates for ex situ plant conservation and propagation (Dutta et al., 2026). Beneficial endophytic bacteria offer a sustainable solution by promoting growth and nutrient uptake. The results provide integrated molecular and ecological evidence that IALR632 promotes plant growth by coordinating host gene expression and restructuring the rhizobiome, offering a mechanistic basis for microbial inoculant strategies in hydroponic horticulture (Amaradasa et al., 2026). Taken together, the obtained data indicate that the application of PGPR improves productivity, although its effectiveness remains dependent on environmental factors, strain selection, and crop management practices (Kolanos et al., 2026).

The structure and abundance of soil microbial communities are strongly correlated with soil physicochemical factors such as organic matter, pH, and the levels of potassium, phosphorus, and nitrogen (Yang et al., 2025). Maintaining the diversity of bacterial and fungal communities in soil mesocosms is essential for sustaining key soil functions (Romero et al., 2026). In soils with a high content of organic matter, microbiological analysis reveals an abundance of *Proteobacteria*, *Candidatus*, and *Saccharibacteria*, which are known for their role in decomposition and nutrient cycling. However, under conditions of high available phosphorus content, *Saprospiraceae* become more prevalent (Qiao et al., 2026). The stochasticity of soil microbes and multitrophic networks was investigated using amplicon sequencing. Soil community functions related to the cycling of carbon (C), nitrogen (N), phosphorus (P), and sulfur (S) were assessed, and dominant bacterial life-history strategies were characterised using metagenomics. Optimisation of intermediate-sized genomes at the microbial community level may enhance taxonomic diversity and increase the abundance of functional genes associated with the C, N, P, and S cycles (Ni et al., 2025).

Increased carbon accumulation in soils can be achieved through carbon sequestration in soil via root biomass and rhizodeposition, including root exudates. The carbon storage potential supports the development of a rich (microbial) soil ecosystem (Turpin et al., 2026). The use of biological preparations in agricultural practice based on nitrogen-fixing microorganisms and PGPR is one of the technological approaches for improving soil fertility and enhancing biological nitrogen accumulation (Hossain et al., 2025). Limited nutrient availability enhanced plant productivity under controlled conditions, suggesting a pathway to reduce fertiliser use in agriculture (Mejia et al., 2025).

Beneficial microbial strains (*Pseudomonas monteilii* and *Bacillus cereus*) were isolated from moss and selected based on key traits (phosphate solubilisation, indole-3-acetic acid production, and siderophore synthesis) that promote plant growth. This system shows strong translational potential for land restoration, sustainable agriculture, and extraterrestrial farming (Park et al., 2025). A systemic mechanism was revealed by which the structure and function of the rhizosphere microbial community interact with plant status, nutrients, and hormones to regulate sugarcane tillering. The findings provide a foundation for microbiome-based strategies to improve sugarcane productivity through integrated management of nutrients, hormones, and microbial communities (Lu et al., 2026). Biotechnological products using structural components of *Azospirillum*, such as flagellin and lipopolysaccharides, have emerged as elicitors that influence host development and defence (Pelagio-Flores et al., 2025).

The rhizosphere microbiome plays an important role in plant growth, nutrient uptake, and overall plant health. A link between the rhizosphere microbiome and plant condition has been identified (Salih et al., 2026). *Microvirga* showed a positive correlation with nitrogen levels but a negative association with the accumulation of micronutrients. These findings suggest that microbial interactions within the rhizosphere influence nutrient homeostasis and plant health (Kwak et al., 2025). Microbial inoculants, as an emerging ecological restoration technology, play a key role in plant growth and nutrient activation in sandy soil regions (Septia et al., 2025). Nine functional microbial strains were selected from three stress environments—sandy soils, coastal saline-alkaline soils, and heavy metal mining areas—for use in the study. The results showed that all inoculants increased plant biomass (Sun et al., 2026). A positive effect of calcium was observed on *Candidatus* and *Nitrosotalea*, while a negative effect was found on *Chloroflexi*, highlighting the importance of considering multiple interacting factors (Subberwal et al., 2025). Phosphorus (P) is the second most important macronutrient required for plant growth, and its availability in most natural soils remains extremely low due to its predominantly insoluble forms. Phosphate-solubilising microorganisms are attracting increasing attention as sustainable and environmentally friendly alternatives to conventional chemical fertilisers (Garcia-Berumen et al., 2025).

However, the identification and characterisation of new strains with enhanced phosphorus-solubilising capabilities remain critical for the development of sustainable agricultural practices. The results indicate that the strain *Paenibacillus* sp. nov. SN-8–1 is a promising biofertilizer candidate with improved phosphorus availability in soil (Jin et al., 2026). Microorganisms increase the bioavailability of nutrients, particularly phosphorus (P), zinc (Zn), and nitrogen (N), while also enhancing plant resistance to drought and salinity. Despite challenges arising from technological limitations and ethical considerations regarding their modification, the integration of PGPR into agricultural practices plays a crucial role in improving soil quality and reducing dependence on chemical fertilisers.

### 4.2 Disease resistance

Soil microflora is an essential component of any agro-phytocenosis, where molecular interactions occur between plants and microorganisms through the exchange of metabolites (Etesami et al., 2026). In the rhizosphere zone, microorganisms generate plant-available nutrients, physiologically active compounds, as well as antibiotic substances that suppress phytopathogens (El-Sharkawy et al., 2026).The rhizosphere microbiome plays a crucial role in plant resistance to soil-borne diseases. However, the characteristics required to explain and predict which microbiota will be effective against soil pathogens are still lacking due to the complexity of soil microbial communities (Bai et al., 2026). Plants dually utilise microbial hormone signalling pathways—either to initiate growth responses or to activate defence responses against pathogen invasion (Tripathi et al., 2025). Biotic stresses such as bacteria, viruses, nematodes, arthropods, and weeds hinder plant development and growth. PGPR enhances plant growth through improved nutrient uptake and protection against pests. *Bacillus subtilis* uses indirect mechanisms to control diseases and protect plants from various pathogens and pests (Wahab et al., 2025).

Host plant factors, including genetic variation, metabolites, and microRNAs, shape the structure and function of the plant microbiome, thereby enhancing disease resistance. However, the application of plant microbiome approaches under field conditions remains limited due to the inherent complexity of plant–microbiome and environment–microbiome interactions (Yin, 2026). Metagenomic analysis showed that biochar selectively enriches beneficial genera (e.g., *Nocardioides*) and saprotrophic *Basidiomycota*, while suppressing the pathogenic *Fusarium*. The integration of nutrient-enriched biochar and PGPR creates a synergistic system that restores soil, supplies nutrients, shapes a functional microbiome, and suppresses pathogens (Peng et al., 2026). The effectiveness of a microbiome-based soil improvement strategy has been demonstrated using the pathogen *Phytophthora nicotianae*, which causes tobacco black shank disease and represents a serious threat to global agriculture (Basu et al., 2026). The coevolution of plant–microbe associations over approximately 450 million years has been a fundamental driver of terrestrial life development, giving rise to mutualistic, commensal, and pathogenic relationships along a dynamic “friend–foe” continuum. Genome editing, synthetic biology techniques, artificial intelligence, and microbiome engineering offer opportunities to improve plant disease resistance through the use of synthetic microbial communities, engineered immune receptors, modulation of plant memory, and microbiome-integrated breeding (Shelake et al., 2026).

Soil microbial diversity modulates the interaction between the pathogen *Bipolaris sorokiniana* and the biocontrol bacterium *Pseudomonas inefficax* in the wheat rhizosphere. Inoculation with *P. inefficax* significantly reduced disease severity caused by *B. sorokiniana*, particularly in soils with low diversity, highlighting its effectiveness in simplified environments where microbial competition is reduced. These findings demonstrate the critical role of soil microbial diversity in shaping the success of biocontrol strategies (Nishisaka et al., 2025).

PGPR exert their beneficial effects in various ways, promoting nutrient uptake and synthesising specific compounds for plants, or preventing and protecting plants from diseases (Vince et al., 2024). Thus, optimising microbial communities serves as a potential mechanism for enhancing crop productivity, improving soil quality, and increasing plant resilience (Marzouk et al., 2025). The implementation of microbiome-based strategies has the potential to reduce reliance on chemical fertilizers and pesticides, thereby supporting sustainable cereal production, restoring soil biodiversity, improving ecosystem health, and ultimately benefiting human nutrition and environmental safety (Cucu et al., 2026).

### 4.3 Detoxification of pollutants

Microbial consortia and rhizosphere-associated taxa accelerate the degradation of pollutants, reduce metal toxicity, and enhance plant resilience in acidic or contaminated soils (Kravchenko et al., 2025). Microorganisms degrade organic pollutants, transform or immobilise metals, and mitigate their toxic effects on plants through chelation, redox reactions, and sequestration, while also supporting soil structure and fertility (Munyaneza et al., 2026). Many bacteria absorb trace amounts of metals through the use of metallophores—small molecules that chelate environmental metal ions (Reitz et al., 2026). Plant–microbe interactions play an important role, as they act synergistically and facilitate the uptake, stabilisation, transformation, and detoxification of heavy metals in contaminated soils (Bhuyan et al., 2026). Microbial diversity can be associated with both the type and concentration of present chemical compounds. Microbial consortia can participate in the degradation of complex pollutants such as polyaromatic hydrocarbons and bisphenols (Muhie et al., 2025). The production of metallophores can be predicted through genome mining, where genomes are screened for homologs of known biosynthetic gene clusters (Shahzad et al., 2025).

Complementing these biochemical processes, quorum sensing (QS) is a regulatory system that modulates microbial cooperation, biofilm formation, and the expression of catabolic genes during degradation (Khan, 2025). Microbial remediation is an environmental restoration method that uses microorganisms to degrade or remove pollutants from soil, water, and other environments. This process exploits the natural metabolic capabilities of microbes to break down contaminants into less harmful or non-toxic substances, or to immobilize them and reduce their bioavailability (Campillo-Cora et al., 2025). Among various pollutants, heavy metals such as cadmium (Cd), lead (Pb), arsenic (As), mercury (Hg), and chromium (Cr) are persistent environmental contaminants that pose significant risks to ecosystems and human health (Mohammad et al., 2025). Microorganisms can interact with these metals through different mechanisms to mitigate their harmful effects, reducing the toxicity, mobility, or concentration of heavy metals in contaminated soils (Shi et al., 2025). Metallorganic pollutants and polymetallic complexes pose a major challenge for removal and environmental restoration. 16S rRNA gene amplicon analysis identified bacterial consortia in these complex wastes, including *Candidatus Nitrosotenuis, Methanosarcina, Bacillus, Nitrospira, Cystobacter, and Flavobacterium. Rhizosphere* samples showed a clear shift toward *Actinobacteria* and *Firmicutes*, indicating a new paradigm for understanding the use of microbes as an effective biotechnological tool for the sustainable restoration of industrially contaminated sites (Iqbal et al., 2026).

Arsenic (As) contamination is a serious problem not only for humans but also for plants. Microorganisms can transform this toxic element and remove it from biogeochemical cycling (Hao et al., 2026a). Phosphate mining soils are contaminated with heavy metals such as lead (Pb) and cadmium (Cd), posing significant environmental risks. Bioremediation of such soils has been studied using modified biochar combined with the phosphate-solubilising bacterium *Bacillus cereus*. Microorganisms, including *Janibacter, Lysobacter, Ornithinimicrobium, Bacillus*, and *Salinimicrobium*, formed the core functional microbiota during soil remediation, showing strong correlations with the immobilisation of Pb²^+^ and Cd²^+^ and with increased levels of available phosphate and organic matter. The genes *zit*B*, czc*D*, znt*A, and *cmt*R were identified as key heavy metal resistance determinants regulating metabolic pathways that stabilise microbial community function following soil remediation in phosphate-mining wastelands (Zhang et al., 2025).

Local *Pseudomonas* isolates from 12 contaminated soils collected in Southern Italy were evaluated to assess their adaptive responses to abiotic stress and their potential for bioremediation. One hundred isolates were obtained and tested for growth under heavy metal stress conditions (Cu, Pb, Cr, Zn, As). The selected strains demonstrated tolerance to multiple metals, with removal efficiencies exceeding 65% for Cr, Zn, Cu, and As, and up to 90% for Pb. The combination of high removal efficiency, stress tolerance, and phylogenetic diversity suggests that these isolates are strong candidates for bioaugmentation in metal-contaminated soils (de Santis et al., 2025). Microbial symbiosis enhances nutrient uptake by plants, highlighting the important contribution of symbionts to phytostimulation under subtoxic contamination conditions (Agathokleous et al., 2025).

The most common explosive organic compounds are trinitrotoluene (TNT), 1,3,5-trinitro-1,3,5-triazinane (RDX), and octahydro-1,3,5,7-tetranitro-1,3,5,7-tetrazocine (HMX). Bacteria such as *Pseudomonas, Bacillus, Klebsiella, Arthrobacter*, and *Acinetobacter* are capable of degrading these compounds. These changes impair essential ecosystem functions such as nutrient cycling and soil functioning, with potential cascading effects on ecosystem stability and resilience (Flores et al., 2025). Petroleum-derived pollutants (gasoline and diesel fuel) pose a significant threat to the soil microbiome. Using microbiological methods and next-generation sequencing (NGS), an increase in P*roteobacteria* and a decrease in the relative abundance of *Gemmatimonadetes, Chloroflexi, Acidobacteria*, *Verrucomicrobia, Planctomycetes*, and fungi were observed. These pollutants stimulated the growth of bacterial genera such as *Rhodanobacter, Sphingomonas, Burkholderia, Sphingobium*, and *Mycobacterium*, as well as fungi belonging to the genus *Penicillium*, while negatively affecting *Kaistobacter, Rhodoplanes*, and *Ralstonia*, as well as fungi such as *Chaetomium, Pseudaleuria*, and *Mortierella*. These findings suggest a potential strategy for the remediation of soils contaminated with petroleum compounds (Borowik et al., 2025).

Soil microbiota plays a crucial role in maintaining ecosystem services such as nutrient cycling, organic matter decomposition, and pollutant degradation. In particular, *Sphaerobacter, Saccharothrix, Actinomadura*, and *Nocardia* were identified as potential dual hosts of both biodegradation genes and albendazole degradation genes (Wang et al., 2026). The ability of soil microbiota to degrade mycotoxins provides an opportunity to preserve soil functionality by promoting intrinsic detoxification processes (Korz and Munoz, 2025). The potential of bacteria to degrade microcystin was analysed. Taxonomic analysis revealed dominant phyla including *Proteobacteria, Actinobacteria, Firmicutes, Chloroflexi*, and *Bacteroidota*. These findings provide new insights into microbial interactions within environmental systems and contribute to the optimisation of pollution remediation strategies (Aba et al., 2026). Biochemical adaptive responses of soil microbiota to pollutants and the role of environmentally friendly remediation processes may contribute to microbial and ecological restoration of soils. Microbiome-based soil restoration is a means of long-term environmental detoxification (Qasim et al., 2025).

Six isolates belonging to *Bacillus* sp.*, Pseudomonas* sp., and *Azotobacter* sp. were tested for pesticide degradation. The highest degradation was observed in the strain *Azotobacter chroococcum* (76A), and its secondary metabolites showed low acute toxicity compared to the parent compound. These results suggest that the *Azotobacter* strain 76A can be used as a primary agent in bioremediation (Chaudhary et al., 2025). In addition, meta-omics approaches such as metagenomics, transcriptomics, and metabolomics provide insights into the active microbial communities involved in rhizoremediation processes (Das and Pandey, 2025). There are four main research directions in current bioremediation trends: determining the mechanisms of interaction between petroleum hydrocarbons and heavy metals in co-contamination systems; investigating the succession of microbial communities during the bioremediation process; utilising biosurfactants to enhance the solubility and biodegradation of pollutants; developing integrative, multi-mechanistic approaches to remediation (Usmonkulova et al., 2025).

In the future, the use of enzyme engineering, QS-based biosensors, artificial intelligence-based modelling, and synthetic QS schemes is becoming a viable tool for optimising bioremediation outcomes (Hao et al., 2026b). This approach outlines pathways for advancing environmentally friendly and sustainable strategies for restoring contaminated soils (Hui et al., 2026). In the coming years, the integration of synthetic biology, omics technologies, microbial electrochemical systems, community dynamics, eco-engineering, and plant-microbe synergy will become pivotal for developing effective and resilient bioremediation strategies.

## 5 Approaches to microbiome engineering

Despite the inherent complexity of core interactions, interdisciplinary approaches consistently provide insights into the functioning and structural principles of the microbiome, which is key to microbiome engineering (Copeland et al., 2025). However, several technological and environmental challenges limit their use in agriculture, and sometimes treatment with plant-beneficial microbes fails to produce the desired results under field conditions (Zhakypbek et al., 2026). Consequently, the development of synthetic microbial communities (SynComs), host-mediated microbiome engineering, or the engineering of transgenic plants capable of expressing desired traits can facilitate plant survival and growth under drought stress (Kumar and Sindhu, 2024). Advances in microbiome engineering—ranging from traditional inoculants to synthetic biology—optimise nutrient availability and enhance resilience to abiotic stresses such as drought (Sharma et al., 2025). While climate change exacerbates these issues, innovations in microbiome research and microbiome-shaping genes (M-genes) offer promising solutions for crop sustainability (Misu et al., 2025).

Inoculation of *Salicornia europaea* with *Brevibacterium casei* EB3 and *Pseudomonas oryzihabitans* RL18 facilitated an increase in biomass production and enhanced immune responses by significantly raising the levels of unsaturated fatty acids, sugars, citric acid, acetic acid, chlorogenic acid, and quercetin. Biopriming induced metabolic reprogramming toward the expression of apigenin, quercetin, formononetin, caffeic acid, and caffeoylquinic acid—metabolites that enhance plant resilience to both abiotic and biotic stress. By highlighting key aspects such as nitrogen fixation, nutrient solubilization, hormone production, and the induction of disease resistance, and by analysing technological advancements related to the use of PGPB—including the identification of effective strains, the development of improved biofertilizers, and genetic engineering—a positive impact on plant growth, development, and health has been demonstrated (Rios-Ruiz et al., 2025). These advancements position PGPR as environmental architects capable of restoring soil health and sustaining sustainable agriculture under environmental stress (Azizoglu et al., 2026). Phytomicrobiome engineering approach includes identifying beneficial microbial strains, optimising their application in agricultural systems, and fostering symbiotic relationships that improve plant development (Sidik, 2026). By understanding the molecular mechanisms that regulate these interactions, scientists can design microbial consortia tailored to specific crops and environmental conditions. Recent developments in phytomicrobiome engineering focus on its goals and applications, as well as the tools and methods used to manipulate microbial communities and enhance plant-microbe interactions (Patyal et al., 2025). Recent advancements demonstrate that microbiome-mediated rhizosphere engineering can mitigate stress by activating specific physiological pathways in the host (Li et al., 2025). Two complementary strategies dominate this field: the design of synthetic microbial communities (SynComs) that combine functional traits to stabilise water-use efficiency, and host-mediated microbiome engineering, which selects adaptive communities through iterative, host-driven filtration (Sa et al., 2025). Further progress will require systematic mapping of microbial functions to plant drought response pathways using multi-omics (Sinha et al., 2025).

Advancements in microbiome engineering strategies—including synthetic biology, microbial consortia design, metagenomics, and CRISPR-Cas—focus on enhancing their practical application in agriculture. The integration of microbiome-based solutions into climate-smart agricultural practices can foster long-term sustainability (Ullah et al., 2025). Recently, microbiome manipulation has emerged as a promising approach to enhancing plant growth by investigating a deep understanding of plant-microbe interactions (Panek et al., 2026). In this context, next-generation sequencing (NGS) methods, omics approaches, and synthetic biology have made significant progress in exploring the plant microbiome and are frequently utilised to investigate the intriguing roles of plant-associated microorganisms. Despite the success of traditional approaches, the application of CRISPR/Cas9, RNA interference (RNAi) technology, rhizosphere engineering, microbiome engineering, and other manipulation methods appears to be a promising strategy for enhancing plant productivity and resilience to biotic and abiotic stressors (Joshi et al., 2025).

Host-mediated microbiome selection is a method of artificial selection for microbiomes that confer beneficial traits to plants. Using a systematic selection protocol that maximises the heritability of microbiome effects, transmission fidelity, and microbiome stability across multiple selection cycles, root microbial communities were developed to provide *Brachypodium distachyon*—a model for cereal crops—with resilience to sodium and aluminium (Pereira et al., 2025). Multi-omics, single-cell, and systems approaches, integrated with CRISPR, metabolic engineering, and artificial intelligence—alongside systems biology driven by both *in vitro* and field studies—support predictive modelling and provide evidence for the evolution of systems-oriented strategies for developing effective bioinoculants (Kumar et al., 2026). Recent innovations focus on leveraging soil microbiomes through novel strategies, such as the application of microbial inoculants—composed primarily of bacteria and fungi—to achieve bioremediation, enhance soil quality and fertility, and even replace traditional inorganic fertilisers (Dmytruk et al., 2026). This integrated approach represents a critical innovation in the pursuit of sustainable food production systems, offering a holistic alternative to traditional, chemically intensive methods (Pandey and Saharan, 2025). A conservative basis for integrating multi-omics with mechanistic and quantitative interpretation can be discerned across genes, transcripts, proteins, metabolites, and transformation products at every level (Cycon, 2026). Key findings suggest that many engineered nanoparticles (e.g., zero-valent iron, biogenic metal oxides) can increase the efficiency of HM immobilisation and contour microbial performance and soil microbiome functions (Kamyab et al., 2025). Emerging advanced technologies, such as microbes engineered through synthetic biology, AI-driven microbial design, circular economy value recovery, and policy management innovations, offer essential elements for building microbial remediation of contaminated soils (Li et al., 2025).

Assessing the functional role of the soil microbiome represents one of the most challenging tasks in environmental microbiology, particularly under conditions of armed conflict. The capability of soil ecosystems to recover, detoxify contaminants, and restore fertility largely depends on the functional activity of bacterial and fungal communities. Military activities disrupt key biogeochemical processes that the microbiome normally performs within healthy ecosystems. Explosions and fuel spills have been shown to suppress the expression and activity of functional genes, such as *nifH*, which are responsible for biological nitrogen fixation. At the same time, environmental stress stimulates denitrification processes, causing microorganisms to release nitrogen in the form of greenhouse gases, including nitrous oxide (N_2_O). The soil microbiome also plays a critical role in the biotransformation of contaminants. Under natural conditions, microorganisms can convert potentially toxic metals into less soluble and less bioavailable forms, thereby reducing their mobility and ecological risk. However, soil acidification resulting from sulfur-containing munitions can alter microbial metabolism and stimulate the production of organic acids. These compounds may increase the mobility and bioavailability of heavy metals, such as lead (Pb) and cadmium (Cd), thereby enhancing their toxicity and facilitating their uptake by plants. Military-related disturbances are also associated with a marked decline in the activity of key soil enzymes, including catalases, dehydrogenases, phosphatases, and proteases. These enzymes are essential for organic matter decomposition, nutrient mineralisation, and the maintenance of soil biochemical processes. Reduced enzymatic activity therefore indicates a loss of soil functionality, limiting its capacity to recycle nutrients and turning it into a biologically inert substrate.

The destruction of mycorrhizal fungi and actinomycetes disrupts the processes responsible for the formation of humic substances. As a result, carbon sequestration is reduced and is replaced by accelerated mineralisation of organic matter, contributing to the loss of fertile topsoil and a decline in long-term soil quality. Significant knowledge gaps remain regarding the functional pathways and genetic mechanisms involved in the complete degradation of complex mixtures of military contaminants, including hexogen (RDX), octogen (HMX), rocket fuel residues such as dimethylhydrazine (heptyl) and melange oxidisers, as well as their transformation products in soils with varying aeration conditions. Bioremediation of petroleum-contaminated soils requires substantial amounts of nitrogen and phosphorus, creating potential competition between microbial communities and plants for available nutrients. At present, there is limited understanding of how to optimise bioremediation practices to ensure efficient contaminant removal while maintaining adequate nutrient availability for plant growth. Furthermore, little is known about how chronic chemical contamination and prolonged mechanical disturbance associated with military activities influence gene expression patterns in soil microorganisms without altering their underlying genetic composition. Determining which microbiome genes are actively expressed at a given time requires the analysis of RNA through metatranscriptomic approaches. However, RNA is highly unstable and can degrade within minutes if samples are not properly preserved. Under wartime conditions, transporting soil samples from frontline areas to laboratories while maintaining the required frozen state is practically hardly realistic. Moreover, the detection of bacterial DNA containing genes associated with the degradation of contaminants such as trinitrotoluene (TNT) does not necessarily indicate that these genes are actively expressed. Metagenomic analyses reveal the genetic potential of microbial communities but not their actual metabolic activity under field conditions. Consequently, the presence of biodegradation-related genes cannot be directly interpreted as evidence of ongoing contaminant degradation. Similarly, measurements of soil respiration, reflected by CO_2_ emissions, and assessments of enzymatic activity are typically conducted under controlled laboratory conditions with optimal temperature and moisture regimes. Such conditions rarely reflect the reality of military-affected soils, where blast waves may have disrupted soil structure, reduced porosity, and caused severe compaction. As a result, laboratory-derived estimates of microbial activity may not accurately represent ecosystem functioning in the field. Another major challenge is the lack of integrated bioinformatic frameworks capable of combining metagenomic, metabolomic, and conventional agrochemical datasets into a unified predictive system. The development of such models would significantly improve the ability to assess soil health, predict ecosystem recovery trajectories, and evaluate the effectiveness of remediation strategies in war-impacted environments.

## 6 Microbiome functions concerning war-related contaminants

At the epicentres of explosions caused by missiles, mines, and artillery shells, temperatures can reach several thousand degrees Celsius. Under such conditions, the living biota in the uppermost and most fertile soil layer is often destroyed. In addition, the mechanical mixing of soil horizons caused by explosions, a process known as bombturbation, buries remnants of aerobic microbial communities at depths where oxygen availability is insufficient for their survival, leading to further microbial mortality (Bonchkovskyi et al., 2025). Heavy metals released from munitions, including lead (Pb), cadmium (Cd), arsenic (As), zinc (Zn), and mercury (Hg), can accumulate within crater soils at concentrations up to twenty times above background levels. Elevated concentrations of these metals exert toxic effects on soil microorganisms by inhibiting essential enzymatic processes, disrupting cellular respiration, and impairing microbial metabolism. Consequently, heavy metal contamination contributes to substantial reductions in microbial biomass, biodiversity, and overall biological activity. Residues of trinitrotoluene (TNT), hexogen (RDX), and rocket fuel components represent potent xenobiotic contaminants in soils. These compounds induce oxidative stress in microbial cells, impair cellular metabolism, and inhibit microbial growth and activity (Palchykov et al., 2025). In addition, combustion products generated from propellants and explosive materials, particularly sulfur and nitrogen oxides, react with moisture to form acidic compounds. The resulting decrease in soil pH is detrimental to nitrogen-fixing microorganisms while simultaneously creating favourable conditions for the proliferation of fungal communities. Under military-induced environmental stress, the soil microbiome undergoes distinct qualitative shifts in community structure and function (**Table 2**). In areas surrounding explosion craters, mineralisation increasingly dominates over humification (Illienko et al., 2026). This imbalance accelerates the decomposition of soil organic matter, resulting in rapid losses of organic carbon and progressive degradation of the soil humus balance.

**Table 2:** Qualitative changes in the microbiota under the military stress.

Groups of microorganisms	Change in abundance	Consequences for the soil
Nitrogen fixers (e.g., *Azotobacter*)	Sharp reduction	The natural enrichment of the soil with nitrogen ceases.
Cellulose-degrading bacteria	Reduction	The decomposition of plant residues slows down.
Spore forms and oligotrophs	Sharp increase	Only bacteria that can sustain in a poor environment survive.
Pathogenic microflora	Potential growth	Accumulation of opportunistic fungi and anaerobic microorganisms (e.g., tetanus and gas gangrene pathogens).

Certain bacterial species and arbuscular mycorrhizal fungi transform mobile (toxic) forms of heavy metals into insoluble forms, thereby immobilising them in the soil and reducing their transfer into groundwater and crops. This process mitigates metal toxicity and limits their entry into terrestrial food chains. The application of specialised microbial consortia, including bioinoculants based on nitrogen-fixing and phosphate-solubilising microorganisms, can artificially restore disrupted microbial processes and accelerate the recovery of soil fertility in agroecosystems. Indeed, the biodegradation of trinitrotoluene (TNT) and hexogen (RDX) relies on specific bacterial strains capable of utilising these toxic compounds as sources of nitrogen, carbon, or energy. However, the degradation mechanisms of these explosives differ substantially. Due to the high stability of its aromatic benzene ring, TNT is most often subjected to cometabolic transformation, resulting in partial degradation and the formation of intermediate metabolites. In contrast, RDX, a cyclic nitramine compound, is generally more susceptible to microbial attack and can be mineralised more efficiently and rapidly by specialised bacterial populations. Trinitrotoluene (TNT) contains a highly stable nitroaromatic ring structure, which contributes to its persistence in the environment. Microbial degradation of TNT is primarily mediated by nitroreductase enzymes that reduce the toxic nitro groups (NO_2_) to less toxic amino derivatives. Among the microorganisms capable of this transformation, *Pseudomonas savastanoi* and *Pseudomonas putida* have demonstrated considerable efficiency under aerobic conditions, reducing TNT nitro groups to aminated compounds and thereby lowering the overall toxicity of contaminated soils. In addition, strains belonging to the genus *Buttiauxella* have emerged as particularly promising TNT degraders. *Buttiauxella* sp. has been reported as one of the most active isolates identified in recent years, capable of metabolising up to 87.5% of TNT within approximately nine hours when supplemented with an additional carbon source.

*Methylobacterium* sp. represents a unique bacterial strain capable not only of transforming TNT but also of partially mineralising it to carbon dioxide (CO_2_), while simultaneously degrading other explosive compounds. *Clostridium nitrophenolicum* is a strict anaerobe that can survive in oxygen-depleted environments, such as deeper layers of explosion craters. Under severe oxygen limitation, this bacterium carries out the deep reductive transformation of TNT, including the cleavage of its aromatic ring structure. The biodegradation of hexogen (RDX) largely depends on the presence of specialised cytochrome P450 enzyme systems in bacteria, particularly those encoded by the *xplA* gene. These enzymes catalyse the initial breakdown of the cyclic nitramine structure by releasing nitrite groups, thereby initiating degradation. *Rhodococcus rhodochrous* is considered a model microorganism for RDX bioremediation. This bacterium harbours a plasmid containing the *xplA* and *xplB* genes, which enable it to utilise RDX as its sole nitrogen source and to completely degrade the cyclic structure of the compound. Other members of the genus *Rhodococcus* are also effective aerobic degraders, rapidly binding, transforming, and detoxifying RDX while producing less harmful metabolic by-products. *Gordonia* sp. is a widely recognised aerobic degrader of nitramine explosives. It is capable of metabolising RDX under nutrient-poor soil conditions, making it particularly valuable for remediation in degraded environments. *Methylobacterium extorquens* has demonstrated a high capacity for mineralisation of RDX, converting the compound into carbon dioxide (CO_2_) and water through microbial metabolic processes. Under real-world wartime conditions, soils are often contaminated with mixtures of TNT and RDX, such as those present in explosive formulations including TG-50 and A-IX-1. Studies have demonstrated that the presence of TNT can significantly inhibit the microbial degradation of RDX. Even at relatively low concentrations (approximately 1 mg L^-^¹), TNT exerts a pronounced toxic effect by suppressing the activity of the cytochrome P450 enzyme system, particularly the XplA monooxygenase, which initiates RDX degradation in strains of *Rhodococcus* and *Gordonia*. As a result, effective bioremediation strategies require the development of microbial consortia rather than the application of individual bacterial strains. Within such consortia, certain microorganisms are responsible for the initial detoxification and transformation of TNT, thereby reducing its inhibitory effects, while other specialised strains simultaneously degrade RDX. Octogen (HMX) is a cyclic nitramine explosive that is chemically closely related to hexogen (RDX), but possesses a larger and substantially more stable ring structure. As a consequence, HMX is generally degraded by microorganisms at a slower rate than RDX and often requires specific anaerobic conditions or cometabolic processes involving supplementary carbon or energy sources. Due to its chemical stability and low bioavailability, HMX has historically been regarded as highly resistant to microbial degradation. However, recent studies have identified microbial isolates capable of degrading HMX through denitration pathways.

*Pelomonas aquatica* and *Kinneretia asaccharophila* are among the most effective contemporary bacterial strains. Laboratory and field-scale studies have demonstrated that these microorganisms can independently remove more than 60% of HMX from contaminated soils within approximately 30 days. Owing to their high degradation efficiency and environmental adaptability, they are currently being explored as components of dry granular biopreparations designed for the remediation of explosive-contaminated sites. *Methylobacterium populum* represents a unique methylotrophic bacterium with an exceptional capacity for HMX biodegradation. This strain is capable of complete mineralisation of HMX, converting approximately 61.4% of the radiolabeled carbon contained within the HMX molecule into harmless carbon dioxide (CO_2_) over a period of 55 days. In addition, *M. populum* can simultaneously transform TNT and RDX. *Planomicrobium flavidum* is an aerobic bacterial isolate recovered from explosive-contaminated soils. This microorganism degrades up to 70% of HMX within 20 days through nitrite elimination pathways, specifically via a single nitrite elimination mechanism operating under cometabolic conditions. *Bacillus toyonensis*, a common soil bacterium, has demonstrated considerable potential for HMX biodegradation at low contaminant concentrations. Experimental studies have reported degradation efficiencies of up to 87.7% within 15 days.

*Pseudomonas* sp. is a specialised denitrifying bacterial strain that has been employed in the treatment of industrial wastewater generated by defence-related facilities contaminated with HMX residues. Under anaerobic conditions, such as those found in deeper soil horizons or sediment layers, HMX degradation may proceed particularly rapidly. In these environments, bacteria utilise reductive pathways that convert nitro groups into nitroso intermediates. The formation of these reduced compounds destabilises the cyclic nitramine structure, leading to spontaneous ring cleavage and subsequent degradation of the molecule.

*Clostridium bifermentans* and other members of the genus *Clostridium* are strict anaerobic bacteria that have been isolated from wastewater treatment systems and sedimentary environments. These microorganisms rapidly transform HMX through reductive metabolic pathways, leading to the formation of methylenedinitramine (MEDINA), a key intermediate metabolite in the degradation process. *Klebsiella pneumoniae*, a facultative anaerobe, degrades HMX under reducing conditions. Its degradation efficiency is enhanced in the presence of a cosubstrate, such as molasses or other readily available carbon sources. *Pseudomonas fluorescens* is notable for its capacity to degrade HMX under anaerobic (anoxic) conditions. This characteristic is relatively uncommon within the genus and highlights the metabolic versatility of the species.

As long as real contamination typically consists of complex mixtures of pollutants, the application of monocultures (single microbial strains) often proves insufficient. Consequently, recent microbiological research has increasingly focused on synthetic microbial communities (SynComs), which combine multiple bacterial strains with complementary metabolic capabilities. Within these engineered consortia, individual strains express distinct key enzymes, including NfsA, YdhA, FdhA, and NirS, which collectively establish an integrated pathway for the extensive degradation of explosive contaminants. In this functional cascade, the first group of bacteria removes toxic nitro groups from the target compounds, the second group cleaves the highly stable cyclic structures of nitramine explosives such as HMX, and the third group mineralises the resulting intermediate metabolites into carbon dioxide and water, restoring ecological balance in polluted soils (Yang et al., 2026).

PGPR serve as key biological protectants and remediation activators in soils affected by polymetallic military contamination. Explosions of munitions, combustion of propellants, destruction of military equipment, and damage to batteries simultaneously release highly toxic concentrations of lead (Pb), zinc (Zn), cadmium (Cd), and antimony (Sb) into the soil environment.

As heavy metals and metalloids are chemical elements and therefore cannot be biodegraded, PGPR function in synergy with plants through two principal remediation mechanisms: phytostabilisation and phytoextraction. In phytostabilisation, microorganisms facilitate the immobilisation of metals within the soil matrix, reducing their mobility, bioavailability, and potential transfer to groundwater or living organisms. In phytoremediation systems based on phytoextraction, PGPR enhance the uptake and translocation of metals into the aboveground biomass of plants, which can subsequently be harvested and safely disposed of.

The mechanisms through which bacteria interact with heavy metals depend on both the specific chemical element involved and the selected remediation strategy.

Lead (Pb) and Cadmium (Cd): Immobilisation (Phytostabilisation). Lead and cadmium are commonly introduced into soils through detonators, shrapnel fragments, ammunition alloys, and other military residues, and are among the most toxic contaminants found in war-affected environments. PGPR reduce their mobility and bioavailability through several complementary mechanisms. One of the primary mechanisms is biosorption by exopolysaccharides (EPS). Bacterial strains such as *Bacillus cereus* and *Pseudomonas fluorescens* secrete extracellular polysaccharide matrices that form a viscous biofilm around microbial cells. The negatively charged functional groups present within these polymers effectively bind positively charged metal ions, including Pb²^+^ and Cd²^+^, converting them into less mobile and less bioavailable forms. As a result, the metals become immobilised within the rhizosphere, reducing their migration into groundwater and their uptake by plants. In addition, these bacteria produce phosphatase enzymes, which release phosphate ions into the surrounding soil environment. The liberated phosphate reacts with lead ions to form pyromorphite, a highly stable lead-phosphate mineral characterised by extremely low solubility and toxicity. The formation of pyromorphite effectively sequesters lead in a chemically inert form, thereby reducing environmental risks and protecting both soil biota and agricultural ecosystems.

Zinc (Zn): Regulation and Phytoextraction. Zinc is commonly introduced into military-contaminated soils through ammunition casings, vehicle components, and certain pyrotechnic formulations. Unlike lead, zinc is an essential micronutrient required for normal plant growth and metabolism. However, at the elevated concentrations typically associated with military activities, zinc becomes phytotoxic and can negatively affect soil microbial communities and ecosystem functioning. Strains such as *Pseudomonas putida* and *Pseudomonas aeruginosa* facilitate the remediation of zinc-contaminated soils through enhanced metal mobilisation and phytoextraction. These bacteria secrete organic acids, including gluconic and citric acids, which lower the pH of the surrounding soil and promote the dissolution of zinc-containing mineral phases. The mobilised zinc can then be absorbed by hyperaccumulator plant species, such as mustard (*Brassica* spp.) or bluegrass (*Poa* spp.), through the transpiration stream.

Antimony (Sb): Specialised Detoxification through Redox Transformation. Antimony (Sb) is widely released into soils as a result of the combustion of detonator primers, tracer ammunition, and illumination rounds. This metalloid represents a particularly challenging contaminant due to its occurrence in different oxidation states with markedly different environmental behaviours and toxicities. In soil environments, antimony predominantly exists in two forms: antimonite [Sb(III)], which is highly toxic and relatively mobile, and antimonate [Sb(V)], which is considerably less toxic and less bioavailable. The environmental risk associated with antimony contamination is therefore strongly influenced by its oxidation state. Specialised PGPR strains, including the recently characterised *Cupriavidus* sp. strain S-8-2, possess the ability to oxidise Sb(III) to Sb(V) through microbially mediated redox transformations. This detoxification process significantly reduces the environmental toxicity of antimony-contaminated soils, with reported reductions in overall toxicity ranging from approximately 50% to 55%. Furthermore, the oxidation of antimonite contributes to the stabilisation of the rhizosphere environment, promotes the development of a more balanced microbial community, and substantially decreases the accumulation of toxic antimony species in the aboveground tissues of plants.

*Cupriavidus metallidurans* is widely regarded as a model organism for heavy-metal bioremediation due to its extensive genetic systems conferring resistance to a broad range of metals, including cadmium (Cd), zinc (Zn), lead (Pb), and antimony (Sb). This bacterium is capable of surviving in heavily contaminated environments where many other microbial species cannot persist.

*Pseudomonas aeruginosa* (metal-remediating strains) produces potent biosurfactants, particularly rhamnolipids, which enhance the desorption of metal ions from soil particles. By increasing metal mobility and bioavailability, these biosurfactants facilitate subsequent uptake by plant roots.

*Bacillus amyloliquefaciens* and *Bacillus subtilis* possess the ability to form highly resistant endospores, allowing them to withstand extreme environmental conditions commonly encountered in war-damaged landscapes, including temperature fluctuations, drought, and nutrient limitation. Their resilience makes them particularly suitable for the inoculation of soils severely disturbed by explosions and military activities. In addition to their stress tolerance, these species contribute to ecosystem recovery and biological nitrogen fixation.

**Figure 3:**
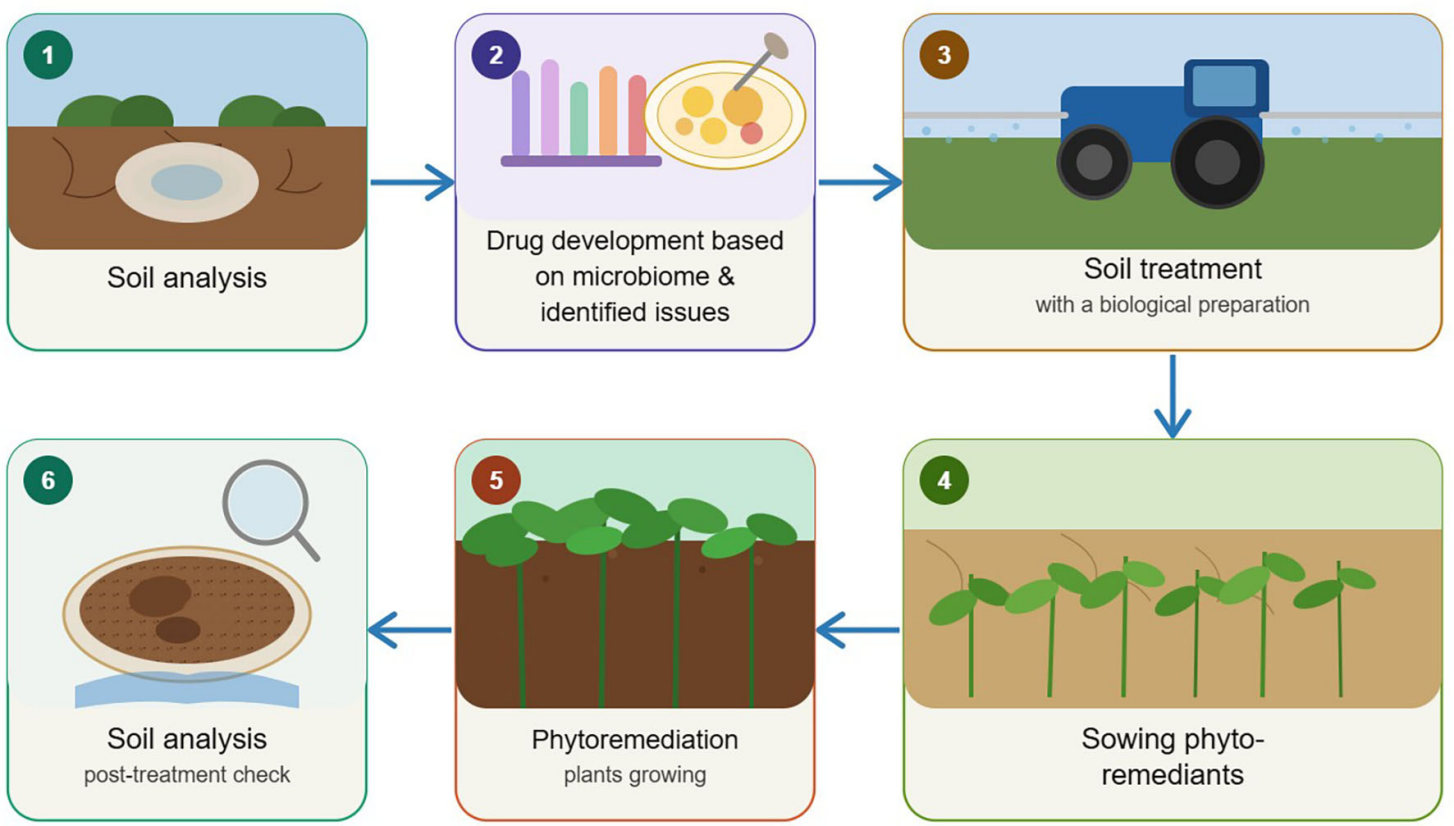
Bioremediation strategy. The visualization was created using Claude Sonnet 4.6.

Bioremediation of soils and aquatic environments contaminated as a consequence of military activities represents an environmentally sustainable approach to ecosystem restoration that utilises microorganisms, fungi, and plants to remove, transform, or immobilise pollutants (**Figure 3**). The principal bioremediation approaches applicable to military-contaminated environments, classified according to the specific type of contaminant, are summarised in **Table 3**.

**Table 3:** Methods of bioremediation of military contamination.

Contaminents	Source of origin	Methods of bioremediation	Main agents (organisms)	Mechanisms of action
TNT, hexane (RDX), tetryl	Explosions of shells, mines, ammunition remnants	Phytoremediation and Microbial Degradation	Plants (*Populus - poplar*, *Typha - cattail*), bacteria (*Pseudomonas*, *Rhodococcus*)	Absorption by roots, cleavage of nitro groups to non-toxic compounds.
Heavy metals (Pb, Cu, Zn, Cd, Cr)	Shell fragments, casings, burnt equipment	Phyto Stabilization and biosorption	Sunflower (*Helianthus annuus*), mustard (*Brassica juncea*), mushrooms (*Aspergillus*)	Accumulation of metals in the aboveground part or their conversion into a form inaccessible to plants in the soil.
Petroleum products (fuel, lubricants)	Fuel spilled from equipment, oil depots destroyed	Biostimulation and Bioaugmentation	Carbohydrate Oxidizing Bacteria (*Acinetobacter*, *Pseudomonas*, *Alcanivorax*)	Oxidation of hydrocarbons to carbon dioxide (CO2) and water (H2O) by the introduction of nutrients or strains.
Rocket fuel components (heptyl)	Missiles falling and shot down	Mycoremediation and Enzyme Biocatalysis	White rot fungi (*Phanerochaete chrysosporium*), specific bacterial consortia	Destruction of toxic hydrazine compounds using extracellular enzymes (laccase, peroxidases).
White phosphorus	Incendiary ammunition	Microbial transformation	Anaerobic and aerobic soil bacteria	Oxidation of toxic white phosphorus (P4) to safe phosphates (PO43-), which are absorbed by plants.

## 7 Knowledge gaps and future directions

Military actions pose significant environmental risks due to the use of weapons of mass destruction, artillery explosions, and chemical contamination, which can have long-term impacts on human health and natural ecosystems (Novakovska et al., 2025). Soils affected by the negative consequences of military operations can be restored and decontaminated through bioremediation (Mytsyk et al., 2025). The choice of rewilding technology depends on the nature and extent of contamination, the intended use or designation of the land undergoing restoration, and the availability of effective and economically viable technologies. It should be noted that the selection of land restoration methods requires a cumulative assessment of the damage level, which includes determining the land-use suitability category, a process that must be based on a reliable study of the soil microbiome (Chukwuneme and Babalola, 2025). Post-war soil restoration requires addressing severe damage, including high toxicity, low microbial biomass, and suppressed microbial activity caused by explosions (Peddle et al., 2025). Leveraging soil microbiomes offers a sustainable, nature-based approach to restoring fertility, improving nutrient cycling, and enhancing plant resilience (Sharma et al., 2025). Perspectives include the use of microbial inoculants and biofertilizers for the restoration of degraded lands, with research focused on microbiome-based solutions for resilient, sustainable post-war agriculture (Romao et al., 2025). The described strategies and approaches for soil restoration and the activation of plant growth and development, based on the integration of microbiome engineering, play a pivotal role in the bioremediation and rewilding of damaged lands (Adeboye et al., 2025).

The transition of synthetic microbial communities (SynComs) from laboratory conditions to open-field environments, particularly within stressed and technologically disturbed ecosystems such as war-affected areas, presents substantial implementation challenges. One of the primary limitations arises from the fact that SynComs are typically developed and evaluated under sterile or semi-sterile conditions, where ecological niches are largely unoccupied and microbial competition is minimal. Following field application, introduced microbial strains encounter highly diverse indigenous microbial communities composed of billions of microorganisms that have evolved and adapted to the specific environmental conditions of the local habitat. Native microbiota exert strong ecological resistance through mechanisms such as antibiosis, competition for carbon sources and nutrients, and competition for colonisation sites within the rhizosphere. As a consequence, artificially introduced SynCom members frequently fail to establish stable populations and may be eliminated within days or weeks after inoculation. SynComs are subject to unpredictable fluctuations (drought, frost, UV radiation).

Furthermore, degraded and war-affected soils are characterised by unique environmental stressors, including extreme acidification or alkalization, elevated concentrations of heavy metals, and residues of xenobiotic compounds such as trinitrotoluene (TNT), hexogen (RDX), and petroleum-based contaminants. Microbial strains that have not been specifically selected or adapted to tolerate such complex stress conditions frequently experience rapid declines in viability following field introduction. In the long term, synthetic microbial communities (SynComs) may also be subject to evolutionary instability. Individual consortium members can accumulate mutations or lose plasmids carrying beneficial functional genes, including those responsible for toxin degradation, nitrogen fixation, or other key ecosystem services. As a result, some strains may evolve into so-called “ecological cheaters,” which consume community resources while no longer contributing to the collective functional performance of the consortium. Moreover, metabolic cooperation (syntrophy) and cell-to-cell communication mediated through quorum sensing are often optimised under controlled laboratory conditions.

In open soil environments, the diffusion dynamics of signalling molecules and metabolites differ substantially from those observed under controlled laboratory conditions. Signal compounds may be rapidly dispersed or washed away by water movement, whereas in compacted soils affected by heavy machinery, their diffusion may be severely restricted. Both scenarios can disrupt microbial communication and metabolic interactions within synthetic microbial communities (SynComs). The loss of spatial organisation and connectivity among consortium members may lead to the fragmentation of SynComs into isolated microbial populations. Individual strains that rely on cooperative interactions for nutrient exchange, detoxification processes, or metabolic complementation often lack the capacity to survive or function effectively when separated from the broader consortium. In addition, SynComs are frequently designed and optimised for specific model plant species or cultivars. Successful establishment within the rhizosphere depends critically on the composition of plant root exudates.

Field crops and wild plant species used for ecological restoration often exhibit root exudation profiles that differ substantially from those of model plants employed during SynCom development. Moreover, the composition of root exudates varies according to plant developmental stage, environmental conditions, and soil characteristics. If a host plant does not release the specific chemical cues required for microbial recruitment and signalling, the introduced SynCom may fail to establish a stable rhizospheric or endophytic community. The large-scale production of SynComs for practical field applications presents additional challenges. Industrial-scale cultivation, often involving fermentation volumes of thousands of litres, is complicated by differences in growth rates among consortium members. During co-cultivation, rapidly growing strains may outcompete or suppress slower-growing partners, leading to significant shifts in community composition and a loss of the intended functional balance of the consortium. Furthermore, the development of suitable formulation technologies capable of maintaining microbial viability during storage, transportation, and field application remains a major technical and economic challenge.

The limited field-scale application of microbial engineering technologies, including both genetically engineered microorganisms (GEMs) and synthetic microbial communities (SynComs), despite their success under laboratory conditions, can be attributed to numerous biological and ecological constraints. In controlled laboratory environments, researchers are able to establish optimal conditions that maximise the expression of target genes and engineered functions. However, these same mechanisms often become inefficient or unsustainable under the complex and resource-limited conditions encountered in natural soils. The synthesis and maintenance of engineered proteins, such as enzymes involved in the degradation of trinitrotoluene (TNT), plastics, or other xenobiotic compounds, as well as proteins associated with enhanced nitrogen fixation, require substantial cellular energy investment in the form of adenosine triphosphate (ATP). In nutrient-poor or war-affected soils, where carbon sources and essential nutrients are often severely limited, the energetic costs associated with maintaining engineered functions can significantly reduce microbial fitness. In contrast, indigenous microbial populations are naturally adapted to local environmental conditions and generally do not allocate metabolic resources to maintaining nonessential engineered traits. Consequently, native microorganisms frequently exhibit higher growth rates and greater competitive abilities, allowing them to outcompete and displace introduced engineered strains through superior metabolic efficiency and ecological adaptation.

Target-engineered genes are commonly introduced into microorganisms via plasmids. Under natural environmental conditions, however, and in the absence of artificial selective pressure, bacteria frequently eliminate these additional genetic elements to reduce metabolic costs and conserve energy. This phenomenon, known as plasmid instability, can result in the rapid loss of engineered traits. Furthermore, elevated mutation rates associated with stressful environments may lead to the inactivation of engineered genes within only a few weeks after field deployment. Soil itself represents an exceptionally complex three-dimensional matrix composed of micropores, mineral particles, organic matter, and air-filled spaces. Consequently, introduced microorganisms often become spatially separated from target contaminants, such as diesel fuel residues, explosive compounds, or heavy-metal hotspots. Without sustained physical proximity to the pollutants, processes such as biodegradation, biotransformation, or bioaccumulation become significantly less efficient or may fail. In addition, the modification of one or several genes frequently produces pleiotropic effects that influence multiple aspects of cellular physiology and metabolism. As a result, engineered microorganisms may exhibit unintended reductions in environmental fitness. For example, strains optimized for enhanced biodegradation or nutrient transformation may become more susceptible to common environmental stressors, including drought, temperature fluctuations, ultraviolet radiation, oxidative stress, or naturally occurring antimicrobial compounds produced by soil fungi.

Healthy soils, and even partially disturbed soils, constitute well-established ecosystems characterised by intense biological competition and complex trophic interactions. Following inoculation, engineered microorganisms frequently encounter predation by native soil fauna, including protozoa and nematodes, which readily consume newly introduced bacterial cells before they can adapt to local conditions or develop protective biofilms. At the same time, resident microbial communities often recognise introduced strains as ecological competitors and suppress their establishment through the production of antimicrobial compounds, including bacteriocins. Even if researchers succeed in developing highly efficient engineered microorganisms capable of extensive soil detoxification or ecosystem restoration, their release into open environments remains legally restricted or prohibited in many countries, including member states of the European Union and Ukraine. One of the most significant risks associated with environmental applications of genetically engineered microorganisms is horizontal gene transfer (HGT). Engineered genetic traits, including genes conferring resistance to heavy metals or encoding specialised degradative enzymes, may potentially be transferred to indigenous microbial populations through natural genetic exchange mechanisms. In particular, the unintended acquisition of such genes by microorganisms pathogenic to humans, animals, or plants could create unpredictable ecological and public health risks.

The economic feasibility of applying synthetic microbial communities (SynComs) and microbial engineering technologies in war-affected regions represents a balance between substantial upfront investments in research and development and the potential for considerable long-term savings in capital expenditures compared with conventional land remediation approaches.

Excavation and soil replacement (conventional remediation): This approach involves the removal of soil contaminated with hazardous chemicals and heavy metals, followed by transportation and disposal in designated landfills or hazardous waste facilities. The cost typically ranges from USD 100–400 per ton of soil. Given the scale of contamination associated with modern warfare, which may affect millions of hectares, this method is economically prohibitive and operationally impractical for large-scale implementation.

Phytoremediation (plant-based remediation): This strategy utilises plants such as sunflower, rapeseed, and poplar to extract, stabilise, or transform contaminants, particularly heavy metals. Although relatively inexpensive (USD 15–50 per cubic meter of soil), phytoremediation is inherently slow, often requiring 5–25 years to achieve meaningful remediation outcomes. Consequently, large areas of agricultural land may remain unavailable for productive use during the restoration period, resulting in substantial economic losses for the agricultural sector.

Synthetic microbial communities (SynComs) and microbial engineering: The application of adapted microbial inoculants can substantially accelerate the degradation of explosive residues, including trinitrotoluene (TNT) and hexogen (RDX), as well as petroleum-derived contaminants. Estimated implementation costs generally range from USD 30–85 per cubic meter of soil, while remediation outcomes may be achieved within 1–3 years. In addition to contaminant removal, microbial technologies offer the possibility of simultaneously restoring biogeochemical cycles.

The principal economic advantage of SynCom-based remediation lies in the multifunctional nature of the consortium. Rather than serving solely as a contaminant-degrading system, synthetic microbial communities can simultaneously incorporate strains capable of biological nitrogen fixation and phosphorus mobilisation. This multifunctionality facilitates the return of degraded land to agricultural production without the need for large inputs of mineral fertilisers, the costs of which often increase substantially during periods of economic instability and supply-chain disruption. Microbial inoculants do not require specialised or highly sophisticated equipment for application. They can be distributed using conventional agricultural sprayers that are already available to most farming enterprises and agricultural producers. Furthermore, microbial formulations can be applied using agricultural drones, which is particularly advantageous for partially mined, inaccessible, or hazardous areas where direct human intervention presents significant safety risks.

Contaminated land may lose up to 80% of its market and rental value, significantly reducing its economic utility and investment attractiveness. The application of microbial remediation technologies has the potential to accelerate the restoration of land productivity and facilitate the revalorisation of agricultural assets owned by local communities, governments, and private landholders.

As noted previously, commercially available laboratory strains often exhibit poor survival and performance under field conditions. Consequently, the most economically viable strategy is frequently the development of native synthetic microbial communities (Native SynComs) composed of microorganisms isolated from the target region. As these microorganisms are already adapted to local environmental conditions, they generally possess a greater likelihood of successful establishment and long-term persistence in contaminated soils. However, the identification and development of Native SynComs require substantial upfront investments before field deployment can begin. In addition, biological remediation strategies carry inherent ecological risks. Environmental stressors such as drought, extreme temperatures, soil acidification, heavy-metal toxicity, or chemical shock may lead to the collapse of an introduced SynCom following application. In such cases, farmers, landowners, or public authorities may lose the entirety of their investment in microbial production, formulation, transportation, and field application, while remediation efforts must be repeated. Another important economic consideration involves logistics and storage. Many engineered microbial strains and specialised microbial formulations require transportation and storage under refrigerated, frozen, or otherwise stabilised conditions to maintain viability and functional activity. Establishing and maintaining such logistical infrastructure in regions affected by warfare, where transportation networks, energy systems, and storage facilities may be damaged or unreliable, can substantially increase the overall cost of implementation.
